# PCDH1, a poor prognostic biomarker and potential target for pancreatic adenocarcinoma metastatic therapy

**DOI:** 10.1186/s12885-023-11474-1

**Published:** 2023-11-13

**Authors:** Xingyi Du, Xiaoyu Yi, Xiaocui Zou, Yuan Chen, Yanhong Tai, Xuhong Ren, Xinhua He

**Affiliations:** 1 Key Laboratory of Structure-Based Drug Design and Discovery, Shenyang Pharmaceutical University, 103 Wenhua Road, Shenhe District, Shenyang, 110016 China; 2 State Key Laboratory of Toxicology and Medical Countermeasures, Institute of Pharmacology and Toxicology, Beijing, 100850 China; 3 State Key Laboratory of Proteomics, Institute of Basic Medical Sciences, National Center of Biomedical Analysis, Beijing, 100850 China; 4 Nanhu Laboratory, Jiaxing, 314002 China; 5 Department of Pathology, No.307 Hospital of PLA, Beijing, 100071 China

**Keywords:** Pancreatic adenocarcinoma, Prognostic biomarker, Protocadherin 1, Flutamide, Pirinixic acid, Therapeutic target, Metastasis

## Abstract

**Background:**

Pancreatic adenocarcinoma (PAAD) is an aggressive solid tumour characterised by few early symptoms, high mortality, and lack of effective treatment. Therefore, it is important to identify new potential therapeutic targets and prognostic biomarkers of PAAD.

**Methods:**

The Cancer Genome Atlas and Genotype-Tissue Expression databases were used to identify the expression and prognostic model of protocadherin 1 (PCDH1). The prognostic performance of risk factors and diagnosis of patients with PAAD were evaluated by regression analysis, nomogram, and receiver operating characteristic curve. Paraffin sections were collected from patients for immunohistochemistry (IHC) analysis. The expression of *PCDH1* in cells obtained from primary tumours or metastatic biopsies was identified using single-cell RNA sequencing (scRNA-seq). Real-time quantitative polymerase chain reaction (qPCR) and western blotting were used to verify *PCDH1* expression levels and the inhibitory effects of the compounds.

**Results:**

The RNA and protein levels of *PCDH1* were significantly higher in PAAD cells than in normal pancreatic ductal cells, similar to those observed in tissue sections from patients with PAAD. Aberrant methylation of the CpG site cg19767205 and micro-RNA (miRNA) hsa-miR-124-1 may be important reasons for the high *PCDH1* expression in PAAD. Up-regulated *PCDH1* promotes pancreatic cancer cell metastasis. The RNA levels of *PCDH1* were significantly down-regulated following flutamide treatment. Flutamide reduced the percentage of *PCDH1* RNA level in PAAD cells Panc-0813 to < 50%. In addition, the PCDH1 protein was significantly down-regulated after Panc-0813 cells were incubated with 20 µM flutamide and proves to be a potential therapeutic intervention for PAAD.

**Conclusion:**

PCDH1 is a key prognostic biomarker and promoter of PAAD metastasis. Additionally, flutamide may serve as a novel compound that down-regulates *PCDH1* expression as a potential treatment for combating PAAD progression and metastasis.

**Supplementary Information:**

The online version contains supplementary material available at 10.1186/s12885-023-11474-1.

## Background

Pancreatic adenocarcinoma (PAAD) is one of the most aggressive forms of gastro-intestinal cancers, with poor prognosis and high mortality rates among patients with solid tumours [1–4]. Pancreatic ductal adenocarcinoma (PDAC) is the most common subtype of PAAD [5]. Pancreatic cancer has gradually become a common cause of cancer-related deaths with a 5 years survival rate of < 10% [6, 7]. Clinical drug therapy, including folinic acid, 5-fluorouracil, irinotecan, and oxaliplatin (FOLFIRINOX) and gemcitabine hydrochloride with nab-paclitaxel (GA), increased the 5 years survival rate for PAAD by only 9% (from 2 to 11%) [8, 9]. Additionally, targeted therapy and immunotherapy have failed to achieve satisfactory results [10]. Despite the recent advent of new treatment approaches for PAAD, surgery remains the only effective modality [11]. There are 37% patients who survive ≥ 5 years after radical surgery [6]. Therefore, it is crucial to identify potential biomarkers for early detection of PAAD. Several recently reported biomarkers have been used for the early detection of PAAD, including PTPN2 and, CerS6; however, these biomarkers do not show sufficiently differential expression between normal and tumour tissues [12, 13]. Thus, it is important to identify new PAAD biomarkers with more effective results.

As the largest subfamily within the cadherins superfamily, protocadherins (PCDHs) not only mediate cell-cell adhesion but also plays a role in neural patterning [14]. Protocadherin 1 (PCDH1), also known as protocadherin 42, was the first PCDHs discovered [15]. Correlation studies shown that common variants of PCDH1 are the susceptibility genes for bronchial hyperresponsiveness, which is a hallmark of asthma [16, 17]. PCDH1 has also been identified as a requirement for cell entry in New World hantaviruses [18]. PCDH1 expression is stringently regulated during the development of other organs and tissues. This suggests a possible role of PCDH1 in some pathogenetic processes, especially in tumourigenesis [19].

In the present study, we investigated the relationship between *PCDH1* expression and PAAD progression using bioinformatics and experimental validation. First, we studied *PCDH1* expression in pan-cancer samples and, compared them with normal samples through data mining using The Cancer Genome Atlas (TCGA) database, and identified the up-regulation of *PCDH1* in patients with PAAD. Furthermore, we investigated the prognostic value of *PCDH1* expression and the potential mechanism underlying its upregulation. Based on the acquired data, it is speculated that the high *PCDH1* expression affects the transforming growth factor-beta (TGF-β) signalling pathway, which is closely associated with tumourigenesis and cancer regulation. Second, we screened small-molecule drugs to reduce the target gene RNA levels and inhibit gene expression to suppress certain tumour phenotypes and improve patient prognosis. Finally, we identified two compounds that significantly reduced the level of target gene RNA and could be used as lead compounds in anti-pancreatic cancer drug research. Our findings suggest that PCDH1 is a promising tissue biomarker, and that these compounds can serve as potential therapeutic agents against pancreatic cancer.

## Methods

### *PCDH1* expression analysis in Pan-Cancer and PAAD

*PCDH1* expression data were procured from TCGA and Genotype-Tissue Expression (GTEx) databases. The R package (version 3.6.3) DESeq2 was used to compare *PCDH1* expression levels between tumours and corresponding normal tissues in the pan-cancer TCGA project. The GEPIA2 platform was used to analyse gene expression based on TCGA and GTEx databases [20, 21]. The “Expression DIY” module was used to visualise *PCDH1* expression data for 179 pancreatic cancer tissues and compare TCGA normal and GTEx data.

### Survival prognosis analysis of *PCDH1* in PAAD

The Kaplan-Meier survival analysis module in GEPIA2 is an online tool used to evaluate the effect of *PCDH1* messenger RNA (mRNA) expression on the prognosis of patients with pancreatic and kidney cancers [20, 21]. These patients were categorised into two groups based on the high and low expression levels of *PCDH1* (50% high *PCDH1* group and 50% low *PCDH1* group). The prognostic value assessment indicators included overall survival (OS) and disease-free survival (DFS) with a 95% confidence interval (CI), hazard ratio (HR), and p-value.

### Diagnostic value and univariate Cox regression analysis of *PCDH1* in PAAD

Receiver operating characteristic (ROC) curves and area under the curve (AUC) were constructed to assess diagnostic value. The ROC curve was analysed and visualised using the R packages ‘pROC’ and ‘ggplot2’, respectively. The AUC under the ROC curve with the corresponding 95% CI was calculated.

A univariate Cox proportional hazards model was used to estimate the HR. The clinical information and pathological parameters of the samples included age, sex, *PCDH1* expression level, and pathological stage of cancer. The R package survival was utilised to perform univariate Cox regression analysis, thereby showing the correlation between genes and cancer. Forest plots were generated using R package ‘ggplot2’.

### Nomogram model construction

A nomogram model was used to predict the prognosis of patients with cancer. The score of each indicator, such as sex, age, or Tumour, Node, Metastasis (TNM) stage, in an independent individual was used to predict the probability of occurrence. *PCDH1* expression was used as an indicator. All scores were summed to generate a total score, with higher scores indicating events that occurred with a higher probability. A nomogram was created using the R package ‘rms’ and was used to evaluate patient survival.

### DNA methylation and micro-RNA (miRNA) database analysis

DNA methylation has been used to investigate the relationship between gene expression and prognosis in patients with pancreatic cancer. MethSurv [22, 23] is a powerful web tool for survival analysis based on clinical data from the TCGA. Prognostic significances of CpG sites were analysed using the ‘Region based analysis’ module in MethSurv. Methylation correlation data for PAAD were obtained using an Illumina Human Methylation 450 BeadChip. Beta values were used to estimate the degree of methylation.

*PCDH1*-related miRNAs were analysed using the miRNA databases miRDB [24, 25] and TargetScan [26, 27]. Both databases use human species as the objects for analysis. Intersection performance and analysis were performed using a VENN diagram [28].

### Protein-protein interaction (PPI) and gene-gene interaction network construction

The protein interaction network of the PCDH1 protein was generated using the Search Tool for the Retrieval of Interacting Genes (STRING) database [29, 30]. The corresponding organism was limited to *Homo sapiens*, and had only the top 50 ranked interactors. The interaction score for confidence was medium (0.400). However, only 29 interacting proteins were mapped in STRING database. To construct the gene-gene interaction network, we used the GeneMANIA prediction server [31, 32]. Fifty genes were short-listed from this site to interact with *PCDH1*.

### Correlation genes analysis of PCDH1

The Correlation Analysis module in GEPIA2 [20] was used to analyse the correlation between PCDH1 and the gene of interest, previously obtained through intersection in PAAD. The correlation coefficient was evaluated using Spearman’s correlation coefficient.

### Gene ontology (GO) and kyoto encyclopaedia of genes and genomes (KEGG) enrichment analysis

GO and KEGG [33, 34] enrichment analysis were performed using R package ‘clusterProfiler’. Interacting proteins and genes from STRING and GeneMANIA were used for the enrichment analysis. All 29 proteins and 59 genes were analysed using GO and KEGG enrichment, respectively. GO enrichment analysis included cellular components (CC), molecular functions (MF), and biological processes (BPs).

### Gene set enrichment analysis (GSEA)

Differential expression data for *PCDH1* in PAAD were downloaded from LinkedOmics [35, 36]. Fifty ‘hallmark’ gene sets were downloaded from the Molecular Signature Database (MSigDB) [37, 38] for GSEA. Enrichment analysis was performed using the R package ‘clusterProfiler’. The R package ‘ggpolt2’ was used to construct the GSEA plots.

### Analysis of *PCDH1* related immune infiltration and immunotherapy

The immune infiltration level in PAAD was obtained using the ‘Gene’ module of TIMER [39, 40]. To determine the abundance of immune cell infiltration in PAAD, six immune cell infiltrates (B cells, CD8^+^ T cells, CD4^+^ T cells, macrophages, neutrophils, and dendritic cells [DC]) were selected.

To further evaluate the immune infiltration score from multiple perspectives, we analysed the RNA-sequencing (RNA-Seq). expression profiles (level 3) and clinical information for PAAD from the TCGA dataset using the R package ‘immunedeconv’. We leverage the unique properties and advantages of the XCell algorithm integrated into the R package. The result of this algorithm was presented through the R packages ‘ggplot2’ and ‘pheatmap’.

Similarly, the expression values of eight genes (*SIGLEC15*, *CD274*, *HAVCR2*, *PDCD1*, *CTLA4*, *PDCD1LG2*, *LAG3*, and *TIGIT*) associated with immune checkpoints from the TCGA dataset were analysed. Heatmap results were presented using the R packages ‘ggplot2’ and ‘pheatmap’.

### Processing of PDAC single-cell RNA sequencing (scRNA-seq) data

Sequence data were obtained from GSE154778 the Gene Expression Omnibus (GEO) datasets [41]. The R package ‘Seurat’ was used for statistical analysis of scRNA-seq data [42]. The primary and metastatic samples were merged into different groups using canonical correlation analysis respectively as different groups. The cell types were obtained using Uniform Manifold Approximation and Projection (UMAP) dimensionality reduction and clustering algorithm among different groups including ductal epithelial tumour cells (ETC), cancer-associated fibroblasts (CAF), tumour-associated macrophages (TAM), epithelial-to-mesenchymal transition (EMT) like-cells, tumour-infiltrating lymphocytes (TIL), DC and endothelial cells (Endo) [41]. Cells types and subtypes were identified previously established cell type markers [43]. COL11A1 and SPARC were used to annotate the CAF; CD14, CD68, and MRC1 for TAM; VWF, KDR and TNFAIP8L1 for Endo; CD3D and CD3G for TIL; KRT19 and EpCAM for ETC; CADM1 for DC; and KIAA0101 [44] and UBE2C [45] for EMT-like cells.

### Cell culture and reagents

The hTERT-HPNE and Panc-0813 cell lines were purchased from Shanghai Fuheng Cell Centre (Shanghai, China). hTERT-HPNE cells were cultured in Dulbecco’s modified Eagle’s medium (DMEM, high glucose) supplemented with 10% foetal bovine serum (FBS). Panc-0813 cells were cultured in Roswell Park Memorial Institute (RPMI) 1640 medium supplemented with 15% FBS and 1× insulin-transferrin-selenium (41,400,045; Thermo Fisher Scientific Inc., Waltham, MA, USA). BxPC-3 cells were purchased from Procell Life Science & Technology Co., Ltd. (Wuhan, China) and cultured in RPMI 1640 medium supplemented with 10% FBS. All cells were cultured at 37 °C in a 5% CO_2_ incubator, and the STR was verified. All media were supplemented with 100 U/mL penicillin and 100 µg/mL streptomycin (Gibco, Grand Island, NY, USA).

Flutamide and pirinixic acid were purchased from Energy Chemical Ltd. (Shanghai, China). Topilotamide and chlormadinone acetate were purchased from MCE (Shanghai, China). Nilutamide was purchased from Topscience (Shanghai, China). The compounds were dissolved in dimethyl sulfoxide (DMSO). The corresponding culture medium was used to dilute the compound solutions, and the cells were treated with these compounds for 24 or 48 h after plating.

### Real-time quantitative polymerase chain reaction (qPCR)

All the cell types (1 × 10^5^ cells/dish) were cultured in 24-well plates. Total RNA was extracted from the cells using TRIZOL reagent. The total RNA was purified using chloroform/ isopropanol, washed with ethanol, and dissolved in RNase free water. Further, real-time reverse transcription (RT)-qPCR was performed using the PowerUp SYBR Green Master Mix (00948467; Thermo Fisher Scientific Inc.). Primers for *PCDH1* (forward, 5’-ACGCCACTCGGGTAGTGTA-3’; reverse, 5’-TCACGGTCGATGGAGGTCTC-3’) and GAPDH (forward, 5’- GGAGCGAGATCCCTCCAAAAT-3’; reverse, 5’-GGCTGTTGTCATACTTCTCATGG-3’) were used for qPCR analysis.

### Western blotting

All the cell types (4 × 10^5^ cells/dish) were cultured in 12-well plates. Radioimmunoprecipitation assay (RIPA) lysis buffer was supplemented with phosphatase and protease inhibitor cocktail and PMSF was used to prepare protein lysates on ice. The protein lysates were then incubated on a shaker at 4 ℃ and centrifuged at 12,000 rpm for 30 min to obtain the supernatant. Total protein was quantified using Quick Start™ Bradford 1× Dye Reagent (5,000,205; Bio-Rad, Hercules, CA, USA) and boiled in sodium dodecyl sulphate loading buffer (105 ℃ for 15 min). Equivalent lysate concentrations were separated using 10% sodium dodecyl sulphate-polyacrylamide gel electrophoresis (SDS-PAGE). Proteins were transferred to a polyvinylidene difluoride (PVDF) membrane and blocked with skim milk in TBST. After washing with Tris-buffered saline with Tween (TBST), the target proteins were detected by overnight incubation at 4 °C with the appropriate primary antibodies (rabbit anti-PCDH1, GTX114620; GeneTex, Irvine, CA, USA). The secondary antibody (111-035-003; Jackson ImmunoResearch, PA, USA) was used to detect the species-specific portion of the primary antibodies by incubating for 1 h at 24 ℃. Electrochemiluminescence (ECL) was used to visualise the protein bands. After washing with TBST, the appropriate reference protein was selected to repeat the incubation process (mouse anti-vinculin and mouse anti-β-tubulin antibodies: Sigma-Aldrich, St. Louis, MO, USA). All the antibodies were used at a dilution of 1:5000. ImageJ software was used to analyse the grey values.

### Immunohistochemistry (IHC)

All the paraffin sections were de-paraffinised using xylene and a gradient alcohol solution. The sections were then repaired using at high-temperature and high-pressure method, followed by blocking with 3% H_2_O_2_. After washing with phosphate-buffered saline with Tween (PBST, 28,352; Thermo Fisher Scientific), the cells were blocked with 5% normal goat serum (16,210,072, Thermo Fisher Scientific). The sections were then incubated overnight with PCDH1 antibody (GTX114620, GeneTex) at a 1:100 working concentration and subsequently with an anti-rabbit secondary antibody (1:400, ZB-2301; ZSGB-BIO, Beijing, China) for 40 min. After nuclear counterstaining with haematoxylin, the sections were dehydrated and sealed with neutral gum. The IHC results were observed under microscope.

### Cell migration assay

Cells were plated in two 12-well plates and cultured until they reached confluent. Confluent monolayers of HeLa and Panc-0813 cells were scratched with a 200-µL pipette tip. After scratching, the plate was washed thrice with sterile saline. The plates were then incubated with the corresponding serum-free medium treated with the test compounds or DMSO. The migrated cells were photographed at 0 and 24 h at the same location. Image J was used to measure the scratch area and calculate the wound recovery ratio using the following formula:

$${\rm{Ratio}}\,{\rm{of}}\,{\rm{wound}}\,{\rm{area}}\,{\rm{recovery}}\,(\% )\,{\rm{ = }}\,\frac{{{\rm{area}}\,{\rm{of}}\,{\rm{original}}\,{\rm{wound}} - {\rm{area}}\,{\rm{of}}\,{\rm{detected}}\,{\rm{wound}}}}{{{\rm{area}}\,{\rm{of}}\,{\rm{original}}\,{\rm{wound}}}}\, \times \,{\rm{100}}$$

### Statistical analysis

GraphPad Prism 8 (GraphPad Software, La Jolla, CA, USA) was used for statistical data analysis. One-way ANOVA was used to analyse statistically significant differences between two groups. Correlations were determined using Spearman correlation analysis. Univariate Cox regression analysis was used to analyse the relationship between clinical characteristics and *PCDH1* expression. For Kaplan-Meier survival analysis curves, p-values were determined from log-rank (Mantel–Cox) test. p < 0.05 was considered statistically significant.

## Results

### *PCDH1* is highly expressed in PAAD

**Fig. 1:**
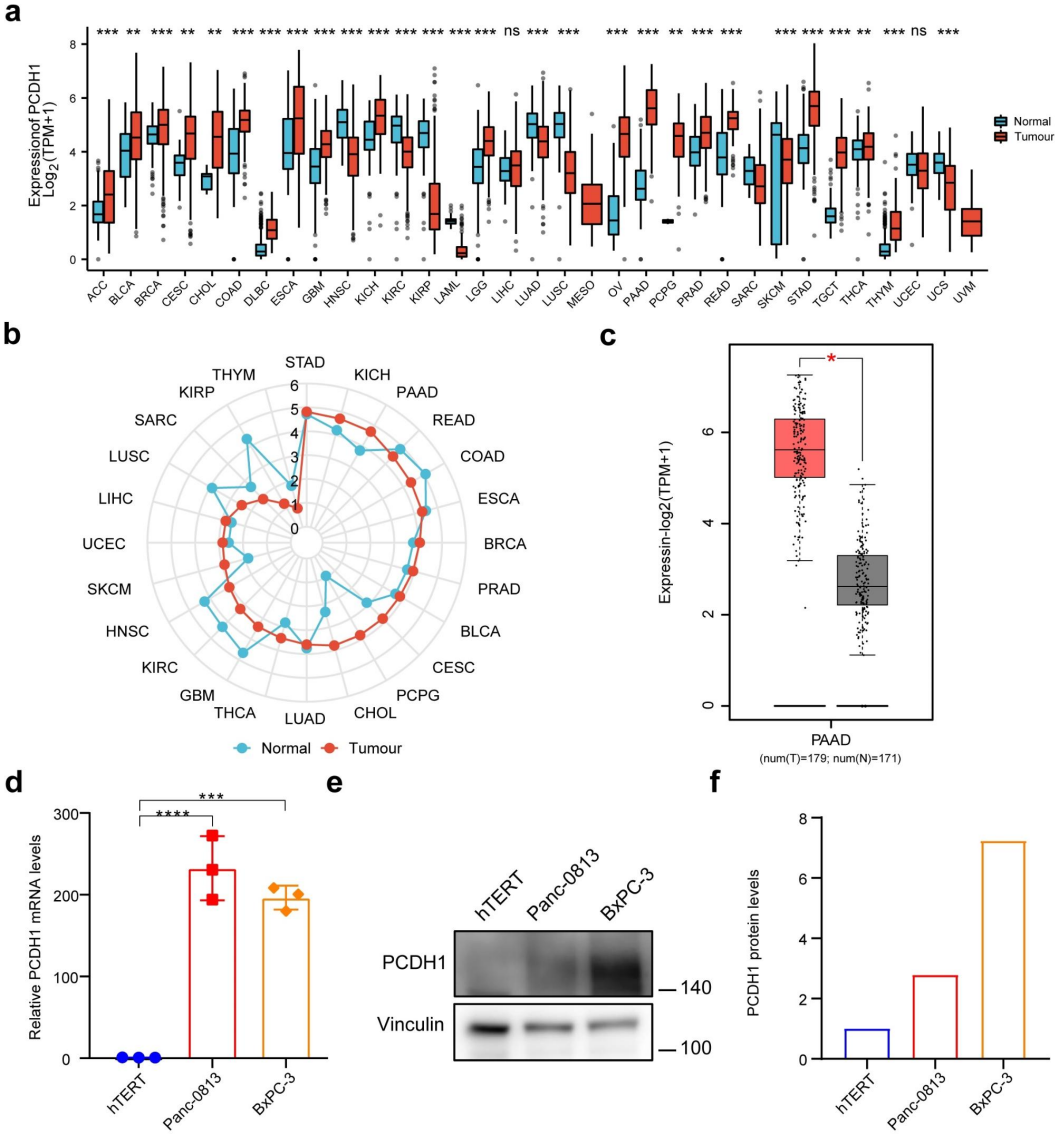
Expression level analysis of *PCDH1* in pan-cancer and PAAD. (**a**) The expression level of *PCDH1* in PAAD was analysed using the TCGA and GTEx samples. Normal sample - blue colour; tumour sample - red colour. (**b**) The level of *PCDH1* mRNA expression in different types of cancers. The radar plot data were shown and compared using the median. The data was sourced from TCGA dataset. (**c**) *PCDH1* expression of PAAD between normal and cancer samples from GEPIA2. Log_2_ (TPM + 1) applied to log-scale. Tumour sample - red colour; normal sample - grey colour. (**d**) mRNA expression level of *PCDH1* in hTERT-HPNE, Panc-0813 and BxPC-3 cells determined using RT-qPCR (n = 3 independent experiments). (**e**) The protein expression level of PCDH1 was detected in hTERT, Panc-0813 and BxPC-3 cells using western blotting. Full-length gels are presented in Additional File 2: Fig. S1. (**f**) The greyscale analysis of (**e**). *p < 0.05; **p < 0.01; ***p < 0.001; ****p < 0.0001

To elucidate the differences in *PCDH1* expression in pan-cancer, expression data were derived from normal GTEx samples and TCGA tumour samples. *PCDH1* was differentially expressed in almost all TCGA cancer types (Fig. 1a). We attempted to analyse the mRNA level of *PCDH1* expressed only in TCGA data. The results display high *PCDH1* expression was identify in PAAD and other cancers, including bladder urothelial carcinoma (BLCA), breast invasive carcinoma (BRCA), cholangiocarcinoma (CHOL), kidney chromophobe (KICH), liver hepatocellular carcinoma (LIHC), pheochromocytoma, paraganglioma (PCPG), prostate adenocarcinoma (PRAD), and thyroid carcinoma (THCA) (Additional File 1: Fig. S1 and Fig. 1b). Notably, *PCDH1* was down-regulated in kidney renal papillary cell carcinoma (KIRP). Moreover, *PCDH1* was up-regulated in pancreatic tumour tissues compared to that in adjacent paired normal tissue samples from TCGA dataset (Additional File 1: Fig. S2). Consistently, *PCDH1* mRNA levels were higher in PAAD tissues than in normal pancreatic tissues, based on the GEPIA2 database analysis (Fig. 1c).

Using existing data, the expression levels of *PCDH1* in PAAD cells were investigated at the cellular level. The results showed that the expression levels of *PCDH1* in the pancreatic cancer cells Panc-0813 and BxPC-3 were significantly up-regulated compared to those in the normal human pancreatic ductal cells hTERT-HPNE (Fig. 1d–f).

### High expression of the PCDH1 gene is significantly associated with poor prognosis in PAAD

**Fig. 2:**
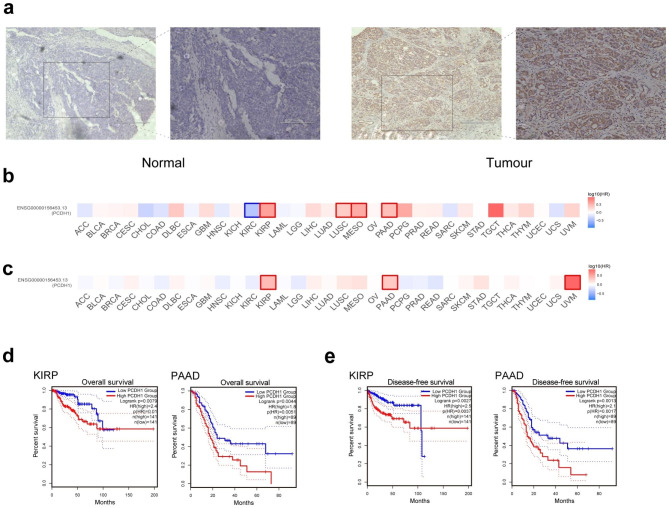
Immunochemical characteristics of PCDH1 and the relationship of corresponding mRNA levels and the prognostic signatures. (**a**) Representative images of PCDH1 IHC staining in pancreatic cancer and normal tissues. Scale bar: 300 and 150 μm. (**b**) OS and (**c**) DFS in prognosis analysis of *PCDH1* expression. (**d**) Kaplan-Meier curve analysis for the association of PCDH1 with OS. (**e**) Kaplan-Meier curve analysis for the association of PCDH1 with DFS in patients of KIRP and PAAD.

After confirming is highly expressed in pancreatic cancer cells, IHC was performed to investigate PCDH1 protein levels in tumour tissue samples of patients, and the results showed that PCDH1 was highly expressed in tumour tissues of patients with pancreatic cancer (Fig. 2a).

Therefore, it was important to determine the correlation between the prognosis of patients with PAAD and the expression level of *PCDH1*, based on which patients were divided into high- and low-expression groups. High levels of *PCDH1* expression correlated with poor prognosis of OS in PAAD and other cancers, including KIRP. Conversely, low levels of *PCDH1* expression correlated with poor OS in KIRP (Fig. 2b). High levels of *PCDH1* expression correlated with poor DFS in KIRP and PAAD (Fig. 2c). Survival analysis was performed using Kaplan-Meier curves to further validate the prognostic value of *PCDH1* transcriptional levels. High *PCDH1* expression was significantly associated with poor prognosis in patients with PAAD (p = 0.0051; Fig. 2d) compared to those with KIRP (p = 0.01). Similarly, the high *PCDH1* expression group exhibited poor DFS in KIRP (p = 0.0037) and PAAD (p = 0.0017; Fig. 2e). High *PCDH1* expression correlates with poor prognosis in patients with PAAD. Taken together, patients with pancreatic cancer with high *PCDH1* expression levels were predicted to have poor OS and DFS.

### High *PCDH1* expression is an independent predictive factor with worse clinical features in PAAD

**Fig. 3:**
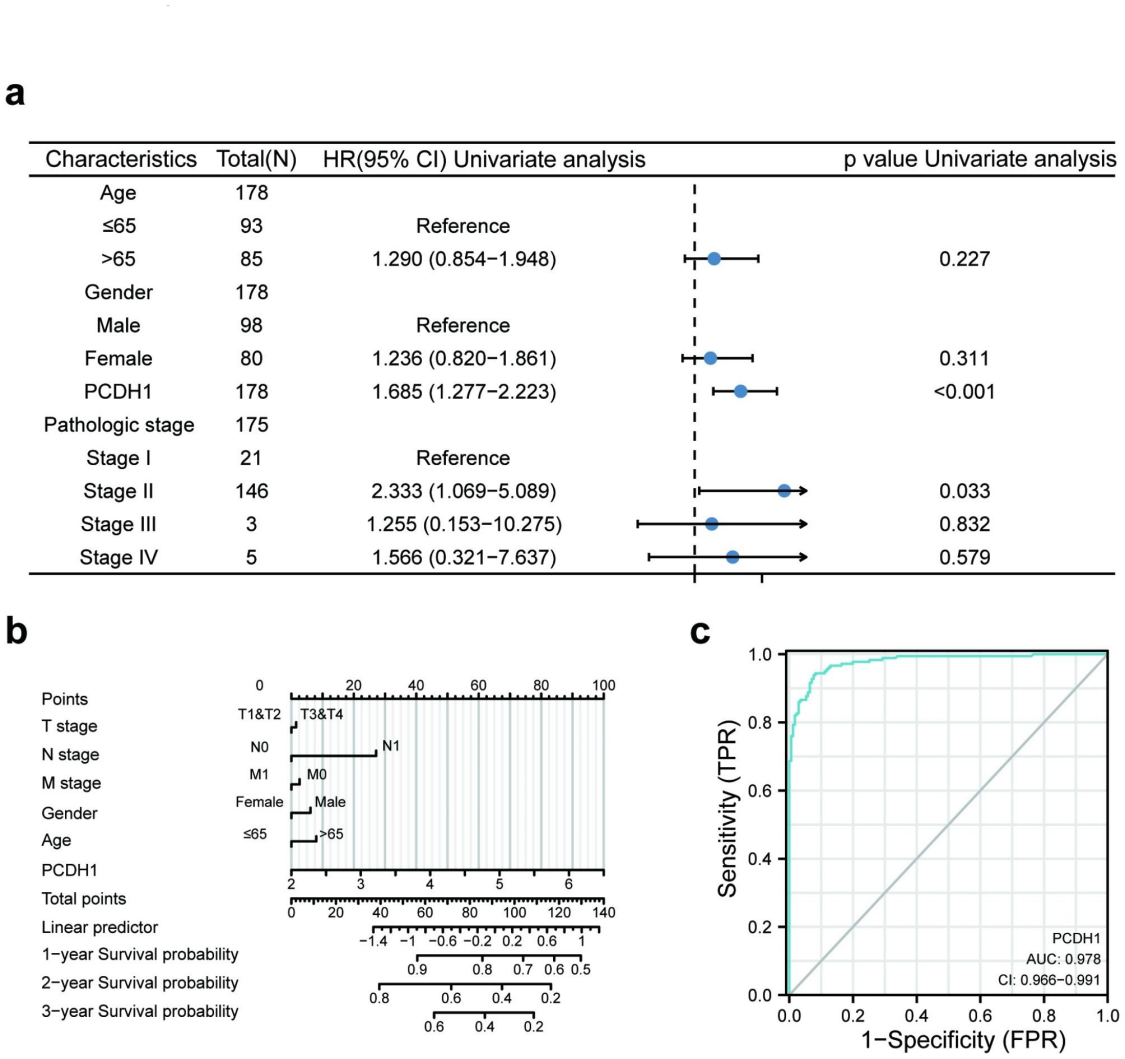
Relationship between PCDH1 expression and the clinicopathological features of PAAD. (**a**) univariate cox regression of risk factors for PAAD. (**b**) The Nomogram prediction model for patients with PAAD. (**c**) ROC curves showing the accuracy of the effect of up-regulated *PCDH1* on the malignant progression of PAAD.

Cox regression analysis was used to examine the relationship between *PCDH1* expression and clinical patients with PAAD using univariate analysis. The expression of *PCDH1* (p < 0.001) and stage II disease (p = 0.033) were the prognostic risk factors for patients with PAAD (Fig. 3a). We constructed a prognostic nomogram to quantify the survival probability of patients with PAAD based on age, sex, T stage, N stage, M stage, and *PCDH1* mRNA levels (Fig. 3b). These results indicated that *PCDH1* expression plays a pivotal role in evaluating the survival probability of patients. The ROC curve was used to evaluate the accuracy of the correlation between gene expression and tumour deterioration based on the AUC. The AUC of the ROC curve for PCDH1 was 0.978, which was used to determine the prognostic value of highly expressed *PCDH1* in PAAD (Fig. 3c).

### DNA methylation, genetic alteration, and miRNA regulate *PCDH1* expression

**Fig. 4:**
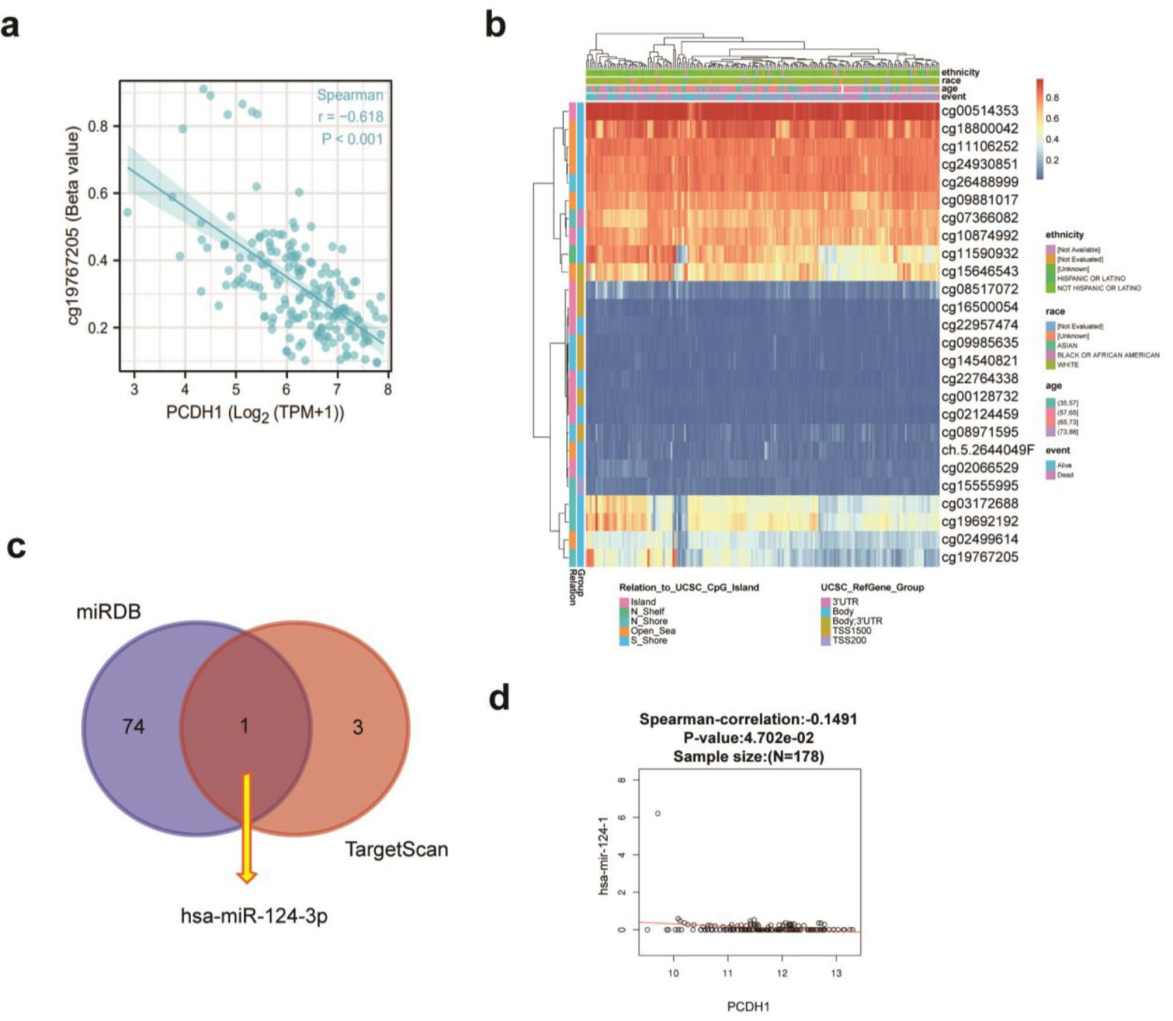
DNA methylation and miRNA regulation of PCDH1. (**a**) Spearman’s correlation of mRNA level of *PCDH1* with methylation cases in key CpG site. (**b**) Methylation heat map of the *PCDH1* methylated probes using the MethSurv database. (**c**) VENN graph of the prediction of miRNAs targeting *PCDH1* in PAAD using the miRDB and TargetScan databases. (**d**) Spearman correlation of mRNA level of *PCDH1* with the target miRNA.

There are several reasons for this high gene expression. The extent of methylation is critical for analysing gene expression. Epigenetic consensus states that DNA methylation is associated with gene silencing [46]. Based on the results of the correlation analysis of the data sourced from the cloud platform, four methylation sites, cg19692192, cg22957474, cg19767205, and cg03172688, showed a significant inverse correlation with *PCDH1* mRNA expression (Fig. 4a and Additional File 1: Fig. S3a). In addition, five methylation sites in *PCDH1*, namely, cg19767205, cg11590932, cg00514353, cg02124459, and cg16500054, were significantly associated with the prognosis of PAAD (Table 1). Consistently, the heat map indicated that a lower degree of cg19767205 methylation correlated with poor prognosis in patients with PAAD (Fig. 4b). In summary, the CpG site of cg19767205 was hypo-methylated and could be a potential cause of the higher expression of *PCDH1*, resulting in a worse prognosis in patients with PAAD.

**Table 1:** The prognostic significances of CpG sites in PCDH1.

CpG Name	Hazard ratio	CI	LR test P value	UCSC Ref Gene Group	Relation to UCSC CpG Island
cg08971595	0.781	(0.482; 1.267)	0.31	TSS1500	S_Shore
cg09985635	1.209	(0.766; 1.91)	0.42	TSS1500	S_Shore
cg14540821	1.187	(0.795; 1.773)	0.4	TSS1500	S_Shore
cg26488999	1.454	(0.889; 2.378)	0.12	Body	S_Shore
cg02499614	0.672	(0.401; 1.124)	0.11	Body	Open_Sea
cg09881017	0.756	(0.462; 1.239)	0.25	Body	Open_Sea
cg11106252	0.721	(0.483; 1.077)	0.11	Body	Open_Sea
cg15646543	1.328	(0.825; 2.138)	0.23	Body; 3’UTR	Open_Sea
cg18800042	1.23	(0.792; 1.909)	0.36	Body	Open_Sea
cg24930851	0.673	(0.407; 1.113)	0.11	Body	Open_Sea
cg03172688	0.642	(0.384; 1.072)	0.077	Body	N_Shore
cg07366082	0.703	(0.443; 1.118)	0.13	3’ UTR	N_Shore
cg15555995	0.813	(0.544; 1.214)	0.31	TSS200	N_Shore
cg19692192	0.744	(0.454; 1.218)	0.23	Body	N_Shore
cg19767205	0.597	(0.36; 0.989)	0.035	Body	N_Shore
cg11590932	0.576	(0.344; 0.964)	0.027	Body	N_Shelf
cg00128732	1.359	(0.822; 2.247)	0.22	TSS1500	Island
cg00514353	0.621	(0.381; 1.011)	0.046	Body	Island
cg02066529	1.269	(0.844; 1.909)	0.26	Body	Island
cg02124459	1.538	(1.026; 2.306)	0.039	Body	Island
cg08517072	1.15	(0.728; 1.816)	0.55	TSS1500	Island
cg10874992	0.684	(0.424; 1.105)	0.11	Body	Island
cg16500054	1.753	(0.994; 3.091)	0.039	TSS1500	Island
cg22764338	1.203	(0.753; 1.922)	0.43	Body	Island
cg22957474	1.259	(0.783; 2.025)	0.33	Body	Island

There were 75 and 4 PCDH1-targeting miRNAs were predicted using the miRNA databases miRDB and TargetScan, respectively. The intersection of the results from the two miRNA databases indicated that the miRNA hsa-miR-124-3p (Fig. 4c). Subsequently, we used LinkedOmics to analyse the correlation between PCDH1 and hsa-miR-124 expression. Only hsa-miR-124-1 showed a statistically significant negative correlation with the mRNA level of *PCDH1* (Fig. 4d and Additional File 1: Fig. S3b). Therefore, hsa-miR-124-1 may regulate *PCDH1* expression.

The alteration frequency of PCDH1 in PAAD was analysed using the cBioPortal website. We identified four samples with alterations in PCDH1 from the mutation and CNA data of 175 patients (PAAD, TCGA, and Pan-Cancer Atlas). These included two (1.14%) cases with amplification and one (0.57%) case each with a mutation and deep deletion (Additional File 1: Fig. S4).

### Identification of related genes interacting with PCDH1

**Fig. 5:**
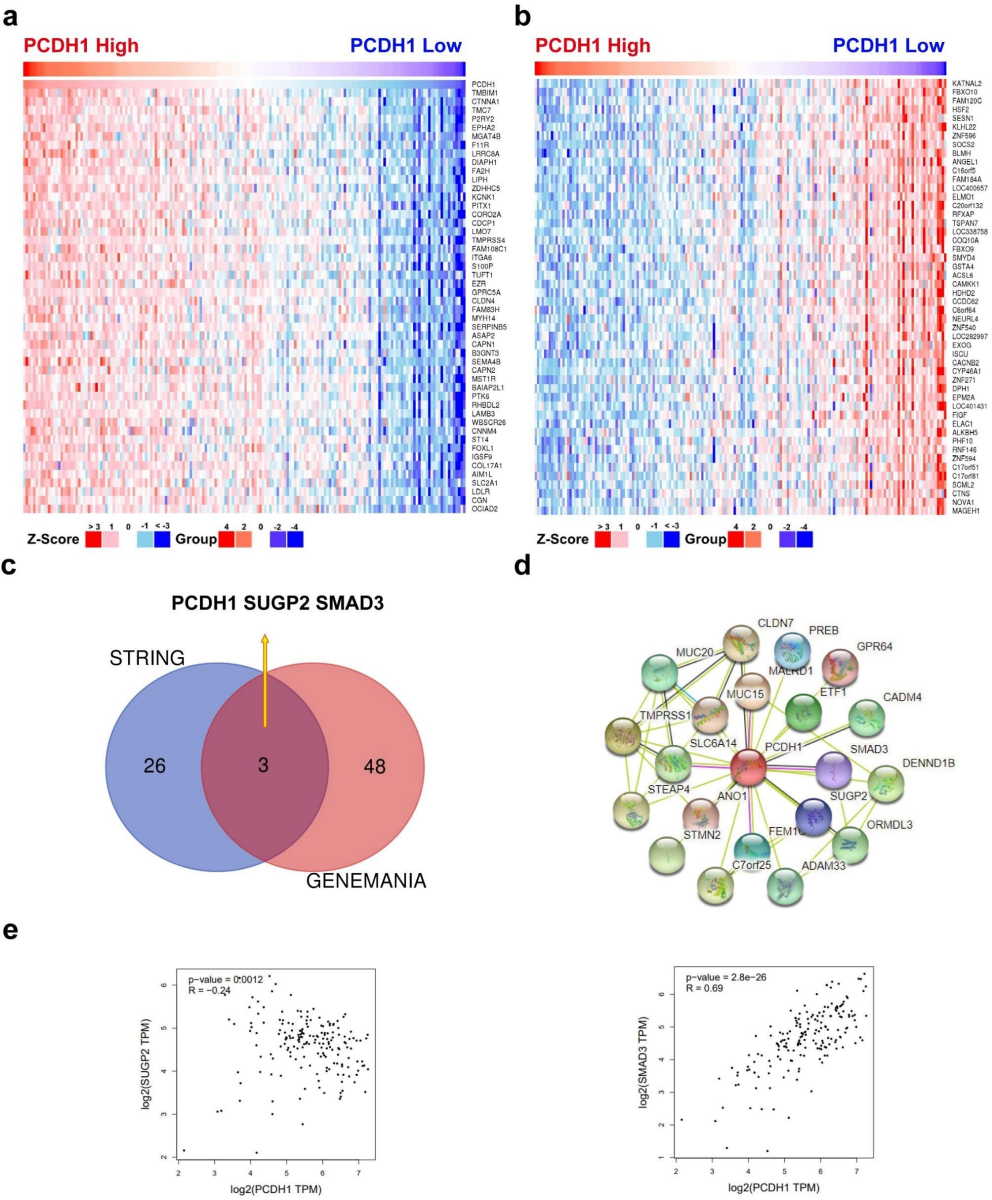
Proteins related to PCDH1 and correlation analysis. (**a**) Heatmap genes positively and (**b**) negatively correlated to PCDH1. (**c**) PPI network map of PCDH1 using STRING. (**d**) VENN graph showing the analysis of the common genes between the STRING and GeneMANIA databases. (**e**) Correlations between the predicted common genes and *PCDH1* expression

To investigate the relationship between PAAD and PCDH1-related genes, we identified several genes closely associated with PCDH1. First, 4524 positively and 7088 negatively correlated genes with *PCDH1* expression in PAAD were analysed. The top 50 genes with positive and negative correlations were ranked by correlation and are displayed in a heat map (Fig. 5a and b). The most significantly positively and negatively correlated genes were *TMBIM1* and *KATNAL2*, respectively. We also predicted the gene-gene interaction network of PCDH1 using the GeneMANIA database (Additional File 1: Fig. S5). The top 29 proteins in the PPI network of PCDH1 were evaluated using the STRING database (Fig. 5c and d). Based on the STRING and GeneMANIA databases, the intersection analysis of these two groups revealed two common members, mothers against decapentaplegic homolog-3 (SMAD3) and SUGP2 (Fig. 5c). Correlation analysis revealed that SUGP2 was negatively correlated with *PCDH1* expression (p = 0.0012, R = -0.24), and SMAD3 was positively correlated with *PCDH1* expression (p = 2.8e-26, R = 0.69) (Fig. 5e). SMAD3 is a key transcription factor mediating the TGF-β signalling pathway, and its receptors are overexpressed in PAAD cells [47]. Moreover, there have been few studies on SUGP2, which indicate that it is mostly involved in RNA processing [48] and spermatogenesis [49].

### The possible mechanisms of PCDH1 promote PAAD proliferation

**Fig. 6:**
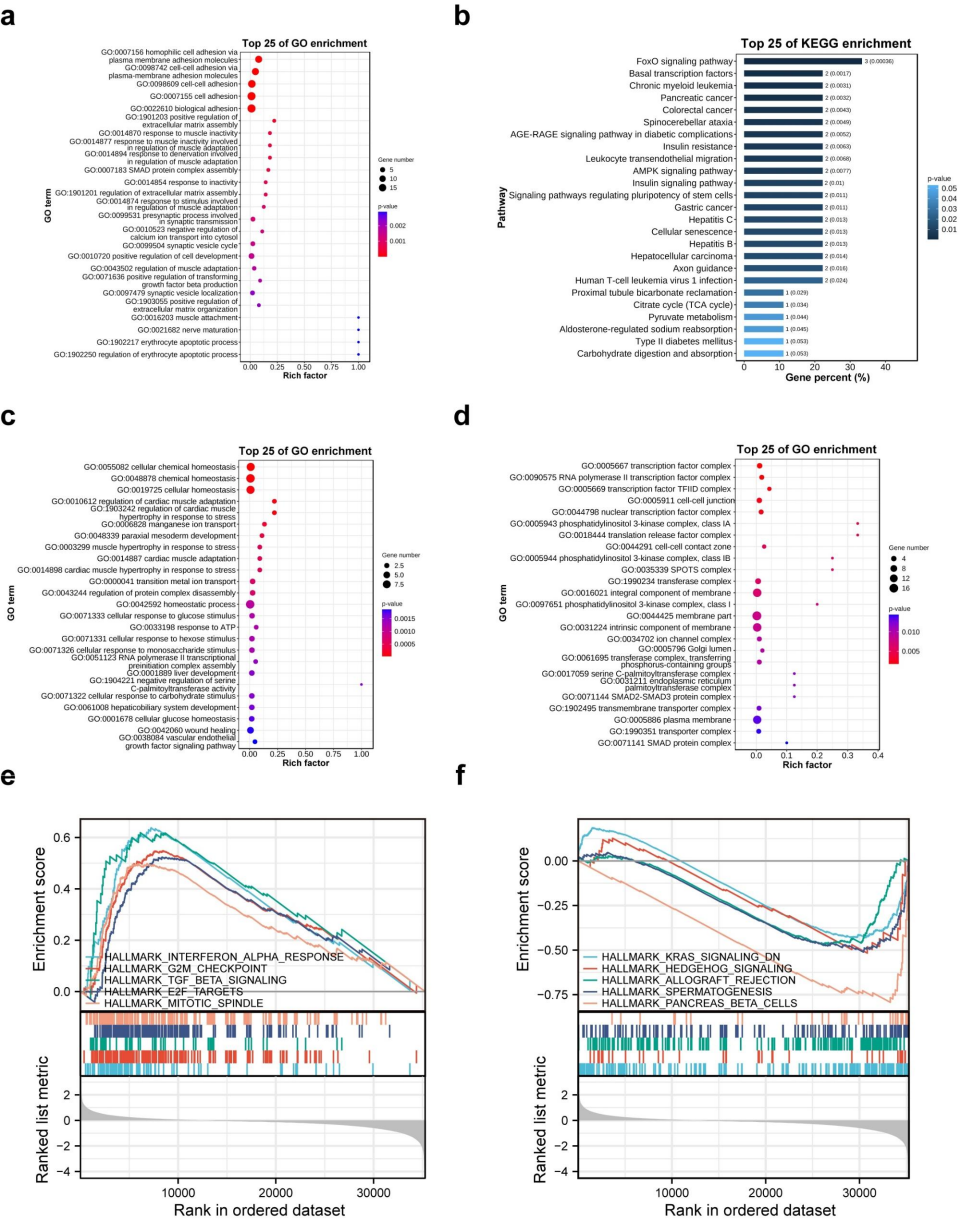
GO and KEGG analysis of *PCDH1* co-expressed genes and GSEA analysis. (**a**) GO (BP) enrichment analysis of PPI genes from STRING. (**b**) KEGG enrichment of PPI genes from STRING. (**c**) GO analysis classified correlated genes from GeneMANIA into the BP and (**d**) CC groups. (**e**) GSEA enrichment plot of the *PCDH1* up-regulated gene sets. (**f**) GSEA enrichment plot of the PCDH1 down-regulated gene sets

GO and KEGG enrichment analyses helped understand the mechanisms by which *PCDH1* promotes PAAD proliferation. The GO enrichment analysis terms were classified into three categories: CC, MF, and BP. Based on the PPI network of PCDH1 from the STRING database, 29 genes were enriched in GO analysis (Fig. 6a and b, and Additional File 1: Fig. S6a and b). The results suggested that most cellular components were associated with the plasma membrane (GO:0044459: plasma membrane part; GO:0005887: integral component of the plasma membrane; GO:0031226: intrinsic component of plasma membrane; and GO:0005886: plasma membrane) (Additional file 1: Fig. S6a). In terms of BP, GO:0005509 (calcium ion binding), GO:0035258 (steroid hormone receptor binding), and GO:0070412 (R-SMAD binding) were markedly enriched among the 29 PAAD genes (Additional File 1: Fig. S6b). Consistently, 29 genes related to cell adhesion were significantly enriched in the MF (Fig. 6a). KEGG analysis revealed that the pathways correlated with 29 genes involved in the FoxO and pancreatic cancer-related signalling pathway (Fig. 6b).

Fifty genes that interacted with PCDH1 were characterised using the GeneMANIA interactive network. Similarly, these genes were used for GO and KEGG pathway enrichment analyses (Fig. 6c and d, and Additional File 1: Fig. S6c and d). Interestingly, *PCDH1*-neighbouring genes were enriched in the transcription factor complex (GO:0005667) in terms of CC (Fig. 6d).

Subsequently, GSEA was used to identify gene sets with consistent differential expression trends. GSEA results showed that high *PCDH1* expression was closely associated with the TGF-β signalling pathway, interferon α (IFNα) response, G2M checkpoint, E2F target, and mitotic spindles (Fig. 6e). Both G2M checkpoint and E2F transcription factors are involved in cell cycle regulation. Proper formation of mitotic spindles ensures the normal progression of cell mitosis [50]. High *PCDH1* expression, which was significantly enriched in the gene sets, may be involved in cell cycle regulation and mitosis.

Low *PCDH1* expression was significantly associated with the KRAS and Hedgehog signalling pathways, allograft rejection, spermatogenesis, and pancreas-beta cells (Fig. 6f). Abnormalities in the KRAS and Hedgehog signalling pathways are associated with tumourigenesis. Mutations in KRAS are prevalent in pancreatic cancer, and hedgehog signalling is an important mediator of pancreatic cancer [51, 52]. The relationship between the low *PCDH1* expression and insulin-secreting pancreatic beta cells is noteworthy.

### *PCDH1* expression is related to multiple immune infiltration cells and immunotherapy

**Fig. 7:**
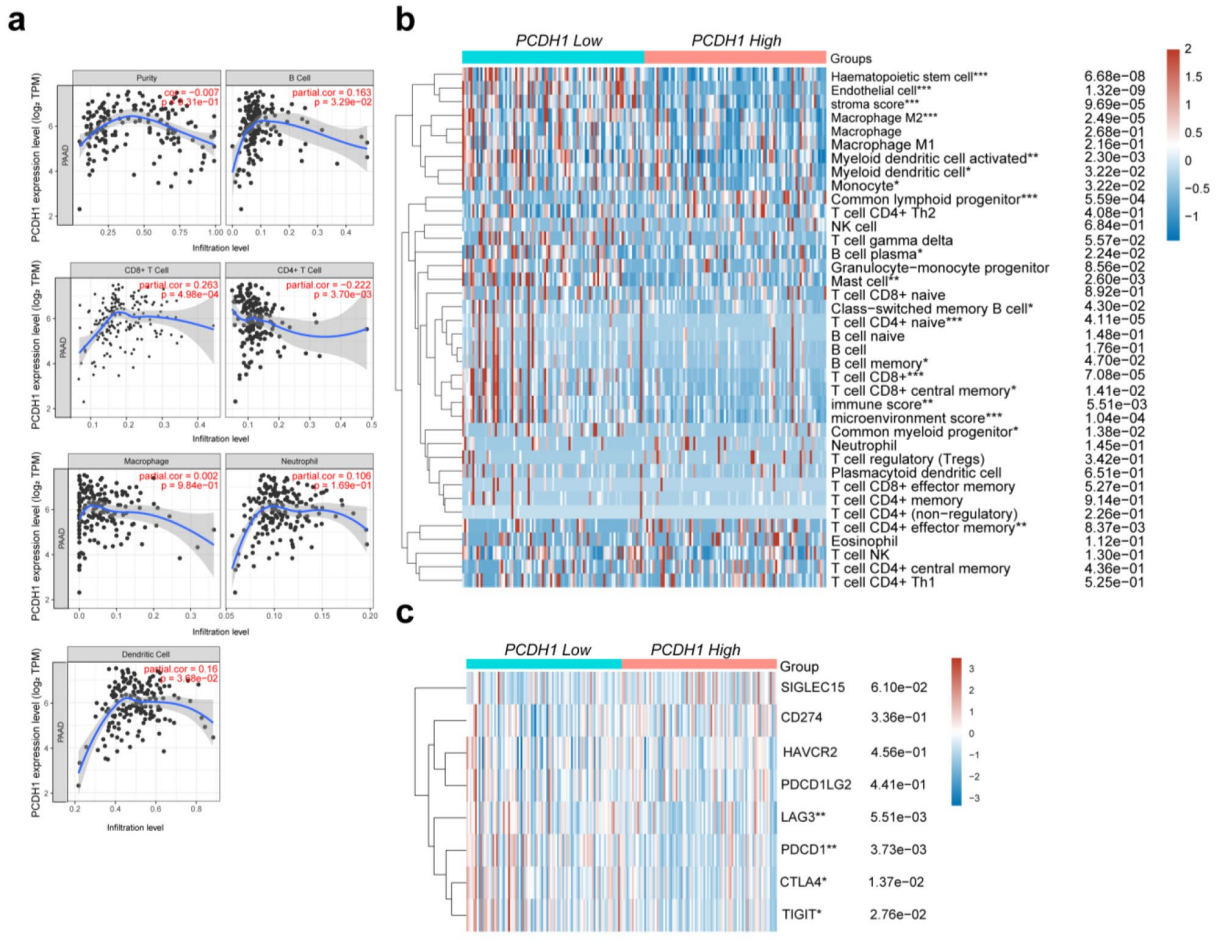
Correlation analysis of *PCDH1* expression with TIICs in PAAD. (**a**) Correlation of *PCDH1* expression with the TIICs based on the TIMER database. (**b**) Heatmap demonstrates the relationship between immune cells score and *PCDH1* expression using xCell. (**c**) The expression levels of four immune-checkpoint genes exhibit significant correlation with *PCDH1* expression in PAAD.

Tumour-infiltrating immune cells (TIICs) are involved in the composition of the tumour microenvironment and have important implications for tumour development and prognosis [36]. The correlation between *PCDH1* expression and six types of infiltrating immune cells, namely B cells, CD4^+^ T cells, CD8^+^ T cells, neutrophils, macrophages, and DCs, was analysed. The results showed that *PCDH1* expression levels were positively correlated with the infiltration of B cells, CD4^+^ T cells, CD8^+^ T cells, and DCs in PAAD (Fig. 7a). The xCell algorithm was used to assess the association between *PCDH1* expression and different immune cell types. The results showed that low *PCDH1* expression was significantly associated with M2 macrophages and CD8^+^ T cells (Fig. 7b). In addition, in PAAD, *PCDH1* expression was associated with immune checkpoint markers including CTLA4, LAG3, PDCD1, and TIGIT (Fig. 7c). These results suggested that PCDH1 plays a role in tumour immunotherapy. This suggests that patients with high *PCDH1* expression should be monitored for immune checkpoints to enable clinical decision-making regarding immunotherapy.

### PCDH1 promotes pancreatic Cancer migration that is inhibited by down-regulation of mRNA expression by compounds

**Fig. 8:**
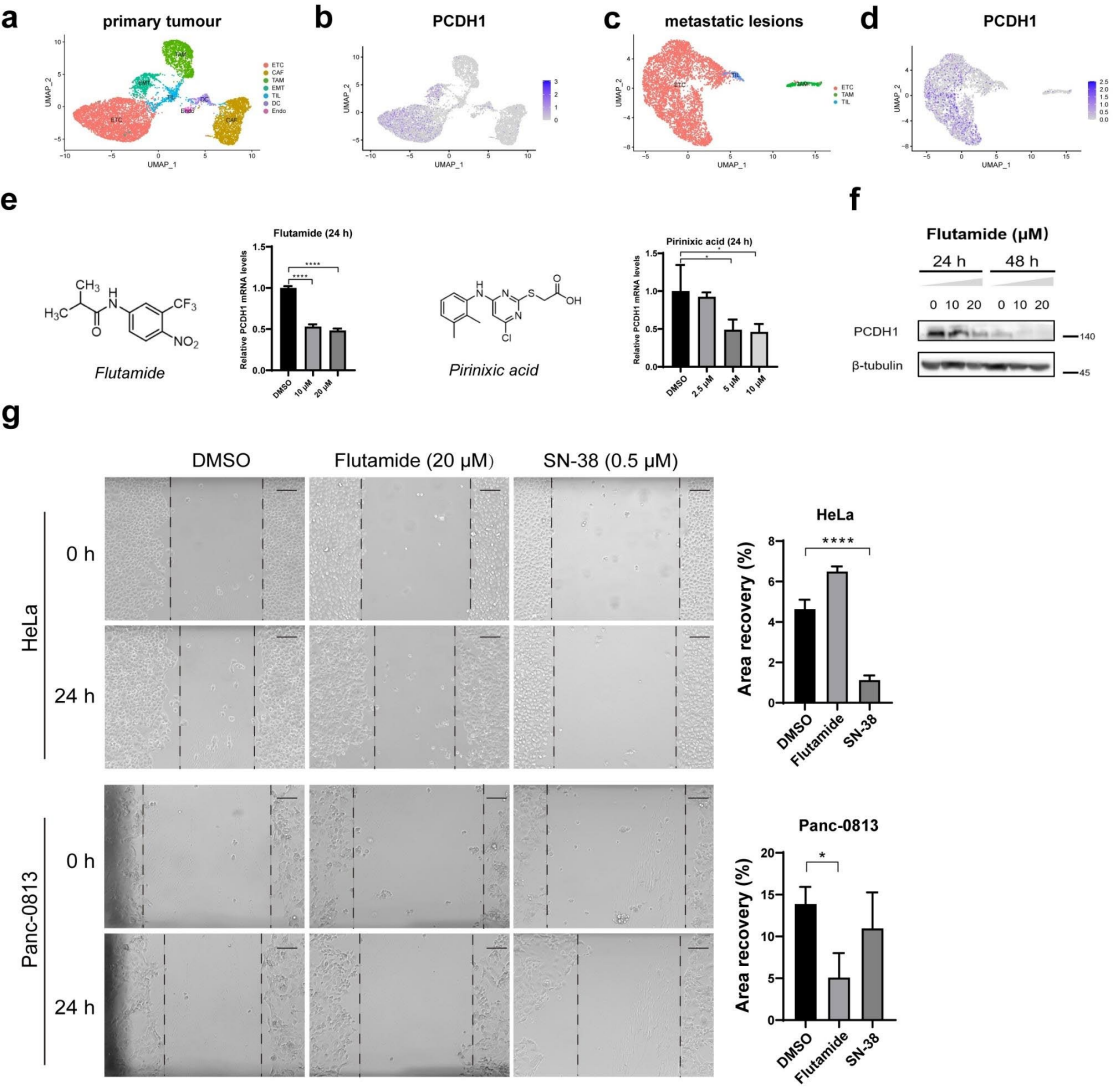
Two compounds reduce *PCDH1* expression in Panc-0813 cells. UMAP visualization of cells from PDAC primary tumour tissues (**a**) and metastatic lesions (**c**). Feature plots showing *PCDH1* expression levels across the cell clusters of PDAC primary tumour tissues (**b**) and metastatic lesions (**d**). (**e**) Inhibition of *PCDH1* expression in Panc-0813 cells at the transcriptomic level (n = triplicate). (**f**) Western blots against PCDH1 protein in Panc-0813 cells treated with various concentrations of flutamide or DMSO. Full-length blots are presented in Additional File 2: Fig. S2. (**g**) A Wound-healing assay of HeLa and Panc-0813 cells treated with flutamide (20 µM), SN-38 (0.5 µM), or vehicle control (DMSO) at 24 h. SN-38 (CAS No. :86639-52-3) was used as a positive drug against HeLa cell migration. Histograms show the change in wound area. Scale bar: 300 μm. *p < 0.05; ****p < 0.0001

Raw sequencing data from ten patients with primary PDAC and six metastatic PDAC lesions from patients with PDAC were used for single cell transcriptome analysis. Both had distinct cell types, including ETC, CAF, TAM, EMT-like cells, TIL, DC and Endo in the primary tissues and ETC, TIL and TAM in the metastatic lesions (Fig. 8a and c). PCDH1 was predominantly expressed in ETC and EMT-like cell clusters (Fig. 8b and d).

To investigate whether PCDH1 is a potential target for pancreatic cancer treatment, several compounds were screened for preliminary exploration. Flutamide and pirinixic acid (Wy-14,643) significantly reduced *PCDH1* expression at the transcriptomic level; however, flutamide was a more potent inhibitor than pirinixic acid (Fig. 8e). Flutamide inhibit androgen receptors (ARs). We also tested other ARs inhibitors, including topilotamide, chlormadinone acetate and nilutamide, for comparison. Not all the inhibitors reduced *PCDH1* expression in pancreatic cancer cells (Additional File 1: Fig. S7). However, the specific mechanisms of action of these compounds remain to be explored. Furthermore, we studied the effects of different flutamide concentrations after 24 and 48 h of exposure. The results showed a concentration- and time-dependent regulation of PCDH1 protein expression in pancreatic cancer cells, as determined using western blotting (Fig. 8f). Next, a wound-healing assay was performed to examine the effect of flutamide on the migratory ability of HeLa and Panc-0813 cells. These results demonstrate that flutamide inhibits pancreatic cancer cell migration at a concentration of 20 µM in vitro (Fig. 8g). In contrast, the same concentration of flutamide failed to inhibit the migration of HeLa cells, which have relatively low *PCDH1* expression [53]. SN-38 was used as a positive control drug for low PCDH1 expression group. Flutamide may be a potential drug for the treatment of pancreatic cancer through the regulation of *PCDH1* expression.

## Discussion

PCDHs are of fundamental biological importance, and some participate in the formation of neuronal circuits and specific synaptic connections [54]. PCDHs are usually identified as candidate tumour suppressor genes; however, some exceptions exist. Protocadherin 7 (PCDH7) promotes the assembly of carcinoma-astrocyte gap junctions, which produces IFNα, which can activate nuclear factor κB (NF-κB) pathways. The loss of protocadherin 8 (PCDH8) expression correlates with breast carcinoma cell progression [55]. However, we found that the overexpression of *PCDH1* was associated with poor prognosis in patients with PAAD, which is specific to PCDH1 [56, 57].

Hyper-methylation is thought to be associated with silencing of tumour-suppressor genes [58]. In this study, during the *PCDH1* gene overexpression in PAAD cells and tissues, the low methylation level of the CpG site of cg19767205 may result in high *PCDH1* expression, which was correlated with poor prognosis. miRNA regulation is another critical step in the expression of target genes that mediate diseases [59]. Candidate hsa-miR-124-1 may regulate the *PCDH1* expression in PAAD cells. However, the specific epigenetic and miRNA regulatory mechanisms underlying *PCDH1* expression require further investigation.

SMAD3 binds to PCDH1, leading to reduced TGF-β-induced gene and protein expression [60]. TGF-β can function either as an early tumour suppressor or as a stimulating factor for tumour invasion and metastasis with tumour evolution. SMAD3 activity has also been implicated in the EMT [61]. Abnormal EMT often enhances cancer cells invasion and stimulates angiogenesis in the tumour microenvironment. We speculated that PCDH1 binds SMAD3 in PAAD cells. EMT may subsequently induce aggressive features and tumour angiogenesis through the TGF-β signalling pathway. Interestingly, scRNA-seq profiling revealed that *PCDH1* is expressed in a cluster, which is a characteristic of EMT. The results of the cell migration assay suggest that PCDH1 play an important role in pancreatic cancer cell metastasis. GSEA results also showed that IFNα responses were significantly associated with high *PCDH1* expression. IFNα is an inflammatory cytokine that acts as a paracrine signal, which activates the NF-κB pathway [62]. PCDH1 interacts with KPNB1 to activate the NF-κB signalling pathway, promoting PDAC progression [63].

The main drug screening strategy was to target *PCDH1* mRNA expression because PCDH1 has been previously identified as a key factor in PDAC progression [63]. In this study, we identified two compounds that can down-regulate the expression of PCDH1. However, PCDH1 down-regulation did not affect the cell viability of pancreatic cancer cells, which is consistent with previous knockdown results [63]. The key role of these compounds is to down-regulate PCDH1 expression and prevent pancreatic cancer metastasis. Flutamide is an ARs inhibitor, and patients taking flutamide survive longer [64]. ARs present in the pancreas can be targeted to treat pancreatic cancer, possibly because testosterone acts as a growth factor in pancreatic cancer [65]. Importantly, flutamide may have new mechanisms for treating, PAAD by inhibiting PCDH1 and not the ARs. Not all the ARs inhibitors down-regulated PCDH1 expression (Additional File 1: Fig. S7). In addition, *PCDH1* expression was down-regulated in pancreatic β-cells (Fig. 6f). Therefore, we speculate that *PCDH1* expression is related to insulin resistance. Panc-0813 is an insulin-dependent pancreatic cancer cell line. The peroxisome proliferator-activated receptor α (PPARα) agonist pirinixic acid improves insulin sensitivity under certain conditions [66]. Interestingly, the potential small compounds specifically down-regulated *PCDH1* mRNA expression, but not protein. Unlike conventional inhibitors, these molecules target RNA, which is a new trend in the development of novel drugs. The next step is to discover the target proteins of these molecules that degrade mRNA.

These novel molecular developments may provide new ideas for the treatment of pancreatic cancer with PCDH1. In subsequent studies, the development of novel molecular probes based on PCDH1 may be helpful for early diagnosis of PAAD. In addition, this study used bioinformatics initially to investigate the underlying mechanism of *PCDH1* expression in PAAD. Further experiments are needed to verify these predictions. For example, pyrosequencing may further quantify the methylation level of *PCDH1* gene in PAAD tissues. Moreover, to investigate the correlation between *PCDH1* levels and EMT, silencing *PCDH1* expression may be used to analyse the expression of the TGF-β signalling pathway-associated genes in PAAD cells. More importantly, the exact mechanism by which flutamide and pirinixic acid down-regulated PCDH1 needs to be elucidated to formulate targeted drug.

## Conclusion

In summary, the TCGA pan-cancer data suggests that *PCDH1* is highly expressed in PAAD. Kaplan-Meier and ROC curves showed that *PCDH1* may be associated with poor prognosis in patients with PAAD. Thus, *PCDH1* could be a potential biomarker for detecting pancreatic cancer and assessing the survival of patients with pancreatic cancer. scRNA-seq analysis and migration inhibition assays demonstrated that PCDH1 expression correlated with pancreatic cancer metastasis. Additionally, we found that some small molecules down-regulated *PCDH1* expression and are potential drugs for the treatment of patients with pancreatic cancer.

### Electronic supplementary material

Below is the link to the electronic supplementary material.

Supplementary Material 1

Supplementary Material 2

## Data Availability

All data supporting the conclusions of this manuscript were available from the corresponding authors.

## List of abbreviations

PCDHs: protocadherins
PCDH1: protocadherin 1
PAAD: pancreatic adenocarcinoma
PDAC: pancreatic ductal adenocarcinoma
TGF-β: transforming growth factor-beta
OS: overall survival
DFS: disease-free survival
PPI: protein-protein interaction
IHC: immunohistochemistry
scRNA-seq: single-cell RNA sequencing
KIRP: kidney renal papillary cell carcinoma
SMAD3: mothers against decapentaplegic homolog-3
CC: cellular component
MF: molecular function
BP: biological process
ARs: androgen receptors
EMT: epithelial to mesenchymal transition
