# Supplementary material for: PCDH1, a poor prognostic biomarker and potential target for pancreatic adenocarcinoma metastatic therapy
Source: BMC Cancer. 2023 Nov 13;23:1102. doi: 10.1186/s12885-023-11474-1 (PMC10642060; doi:10.1186/s12885-023-11474-1)

**Fig. S1. The expression level of *PCDH1* in PAAD was analysed by the TCGA tumour and adjacent normal tissues samples.**

**Fig. S2. Pan-cancer analysis of *PCDH1* in paired samples.**

**Fig. S3. DNA methylation and miRNA regulation of *PCDH1*.** (a) Spearman correlation of mRNA level of *PCDH1* with methylation cases in different CpG sites. (b) Association of other chromosomally processed miRNAs with PCDH1.

**Fig. S4. Mutation feature of *PCDH1* in PAAD.**

**Fig. S5. Gene-gene interaction network using GeneMANIA**.

**Fig. S6. GO and KEGG analysis of *PCDH1* co-expressed genes and GSEA analysis.** GO analysis classified PPI genes from STRING into CC(a) and MF(b) group. (c) GO(MF) enrichment analysis correlated genes from GeneMANIA. (d) KEGG enrichment of genes from GeneMANIA.

**Fig. S7.** **Effect of ARs inhibitors on *PCDH1* mRNA expression.** Topilutamide and nilutamide exhibited no effect on *PCDH1* mRNA expression (n = three independent experiments). Flutamide-treated groups was used as positive control.

**Fig. S1.**


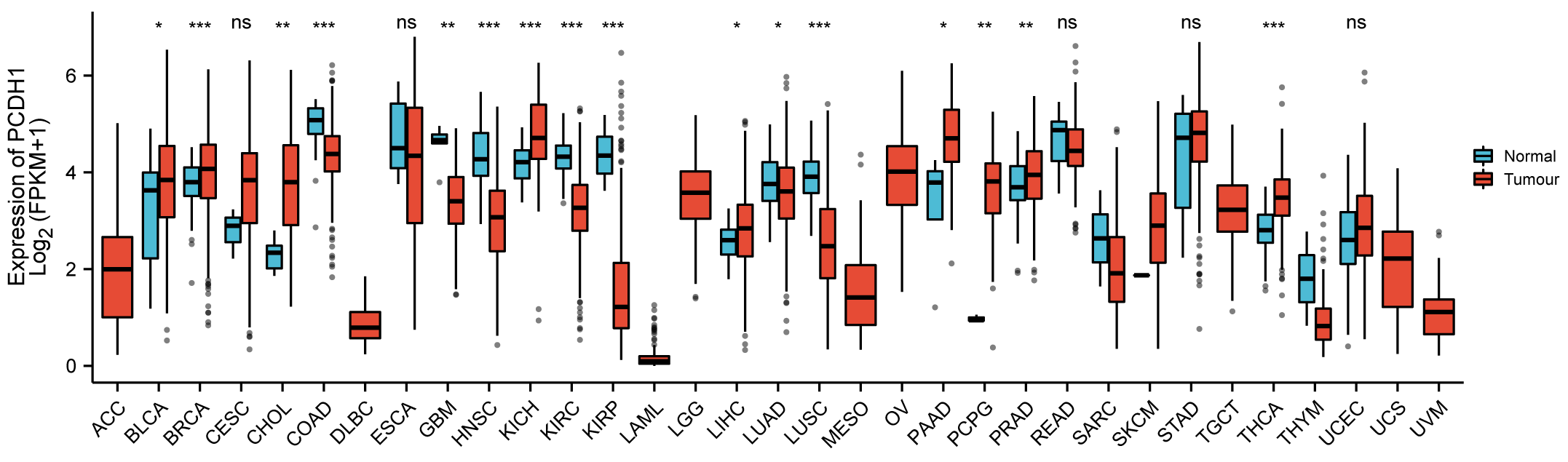


**Fig. S2.**


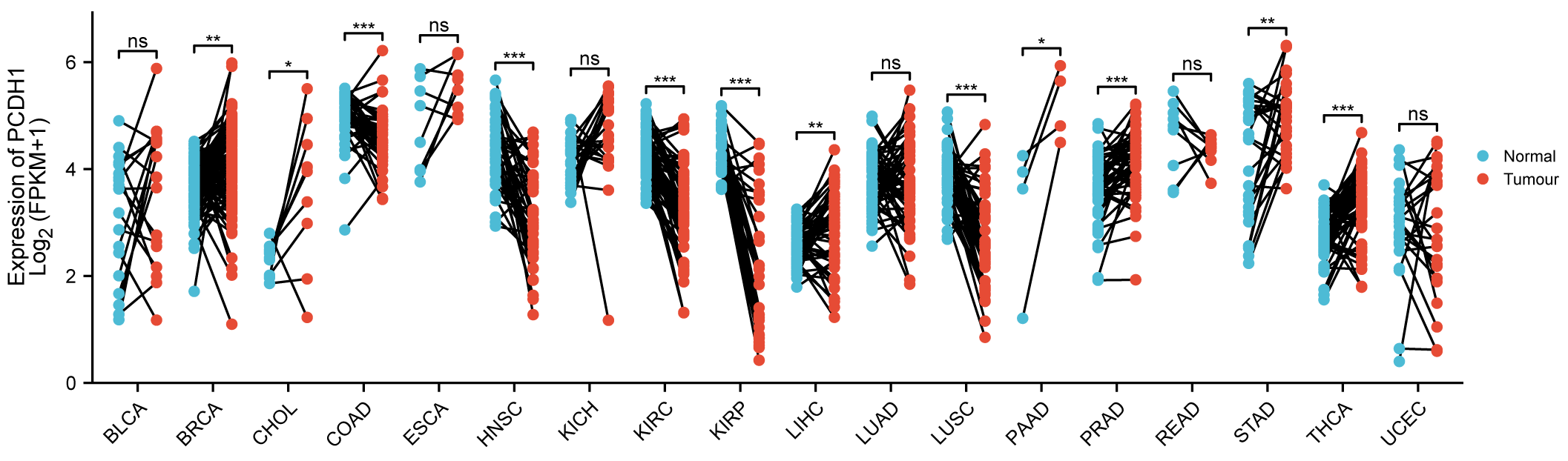


**Fig. S3.**


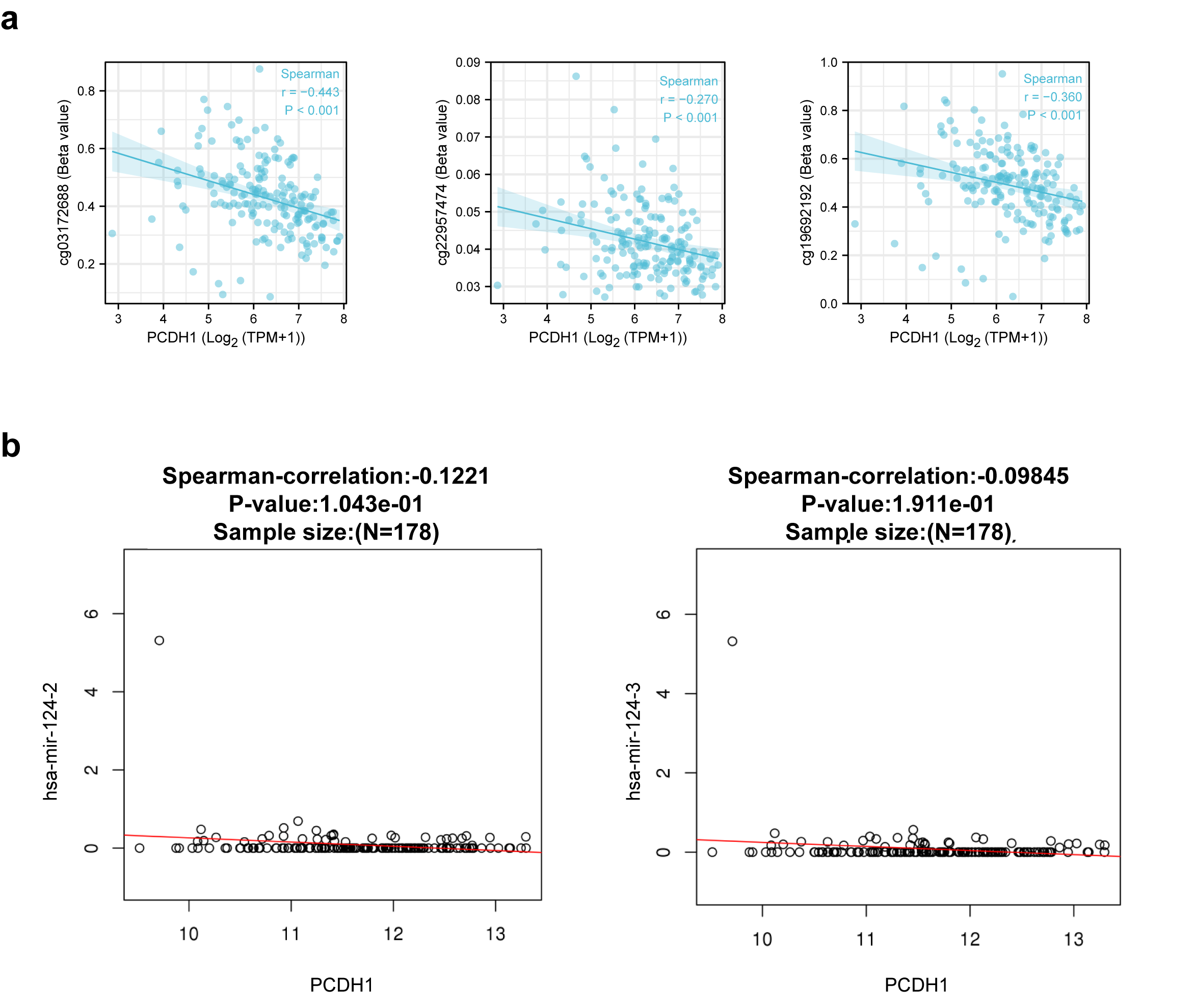


**Fig. S4.**


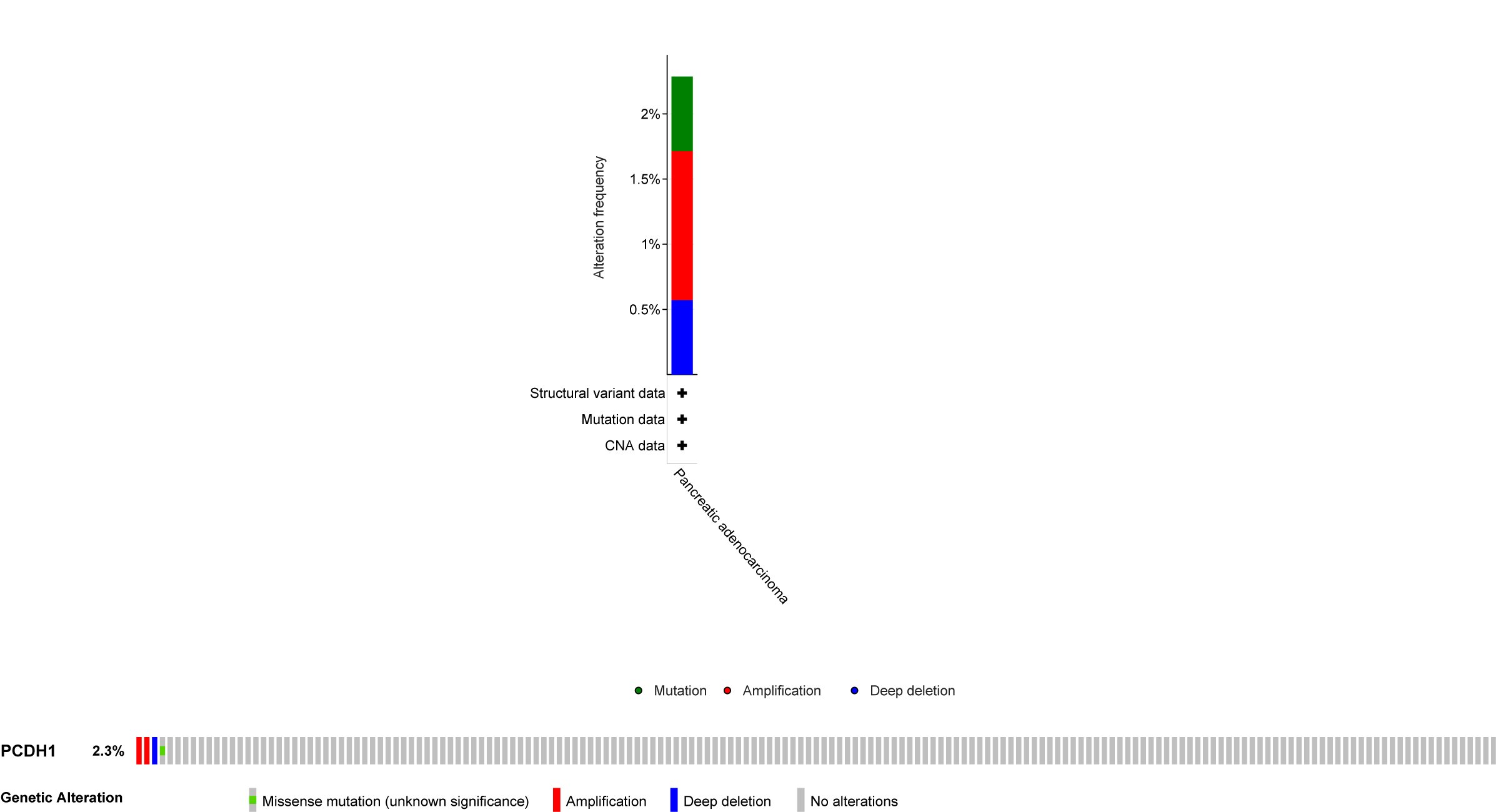


**Fig. S5.**


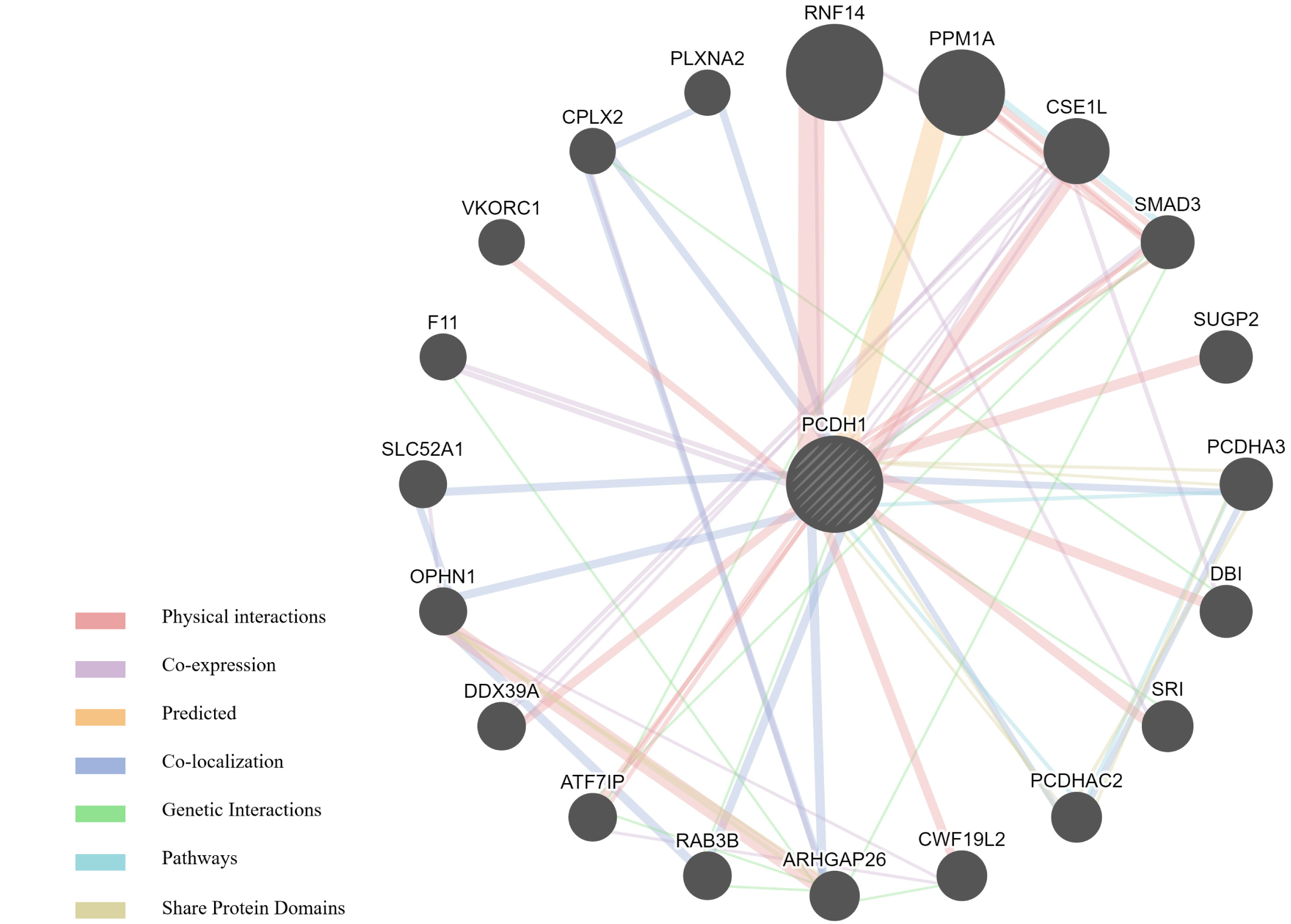


**Fig. S6.**

**
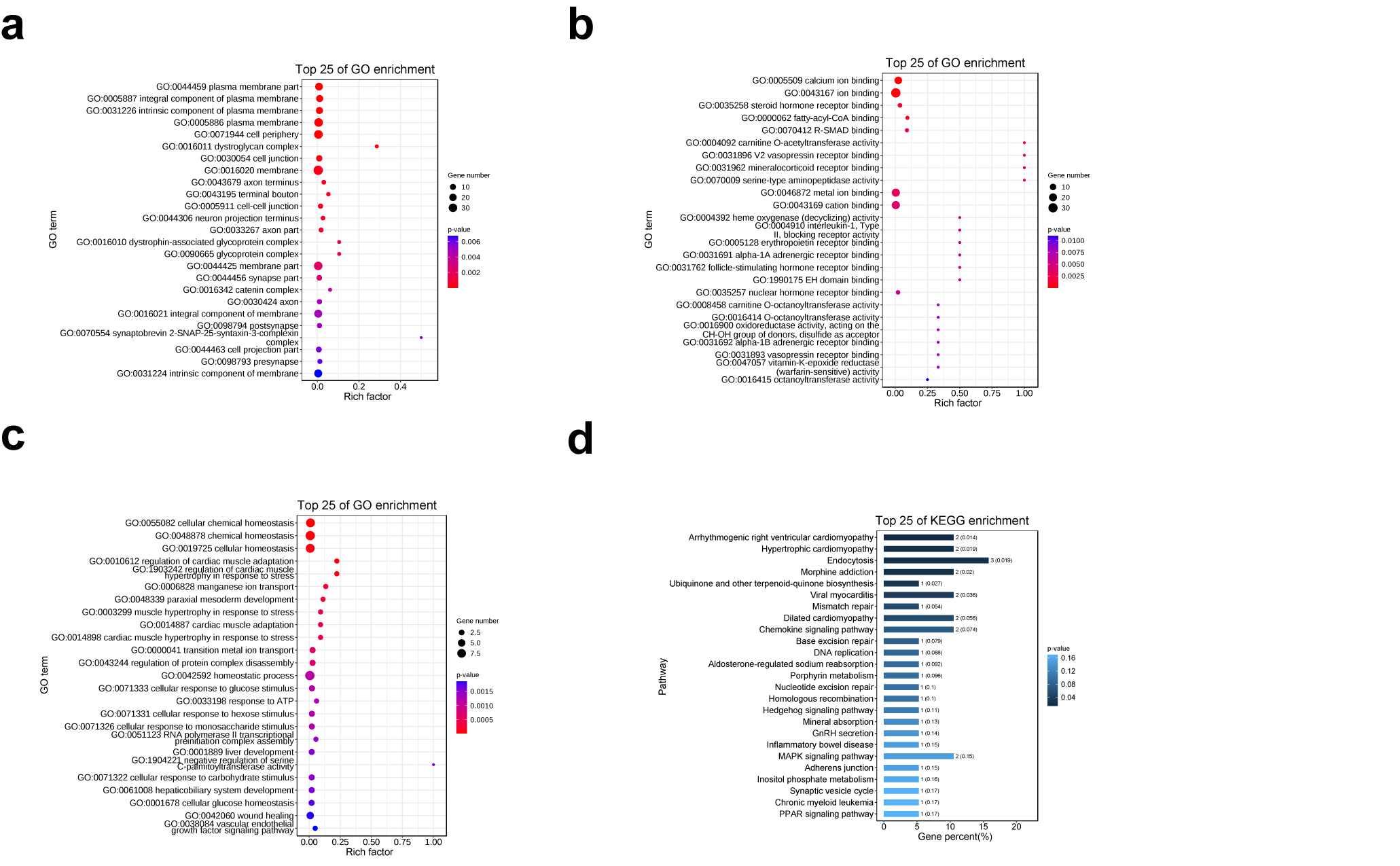
**

**Fig. S7.**


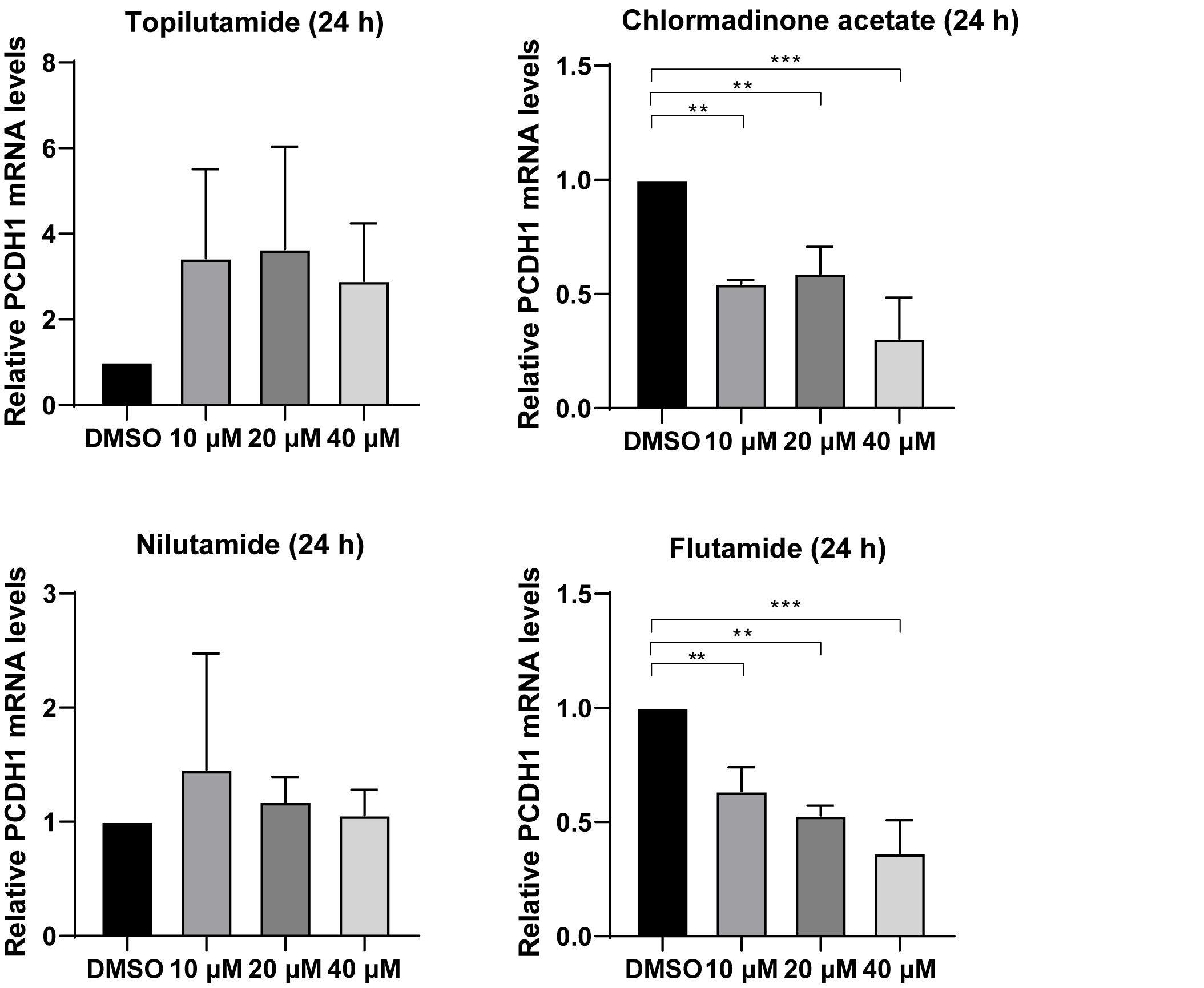

Supplement: Supplementary file 1 — Supplementary Material 1 [file 12885_2023_11474_MOESM1_ESM.docx]
