# Supplementary figures and images for: PCDH1, a poor prognostic biomarker and potential target for pancreatic adenocarcinoma metastatic therapy
Source: BMC Cancer. 2023 Nov 13;23:1102. doi: 10.1186/s12885-023-11474-1 (PMC10642060; doi:10.1186/s12885-023-11474-1)

**Fig. S1. Full-length gels for Fig.1e.**

**Fig. S2. Full-length gels for Fig.8f.**

**Fig. S1.**


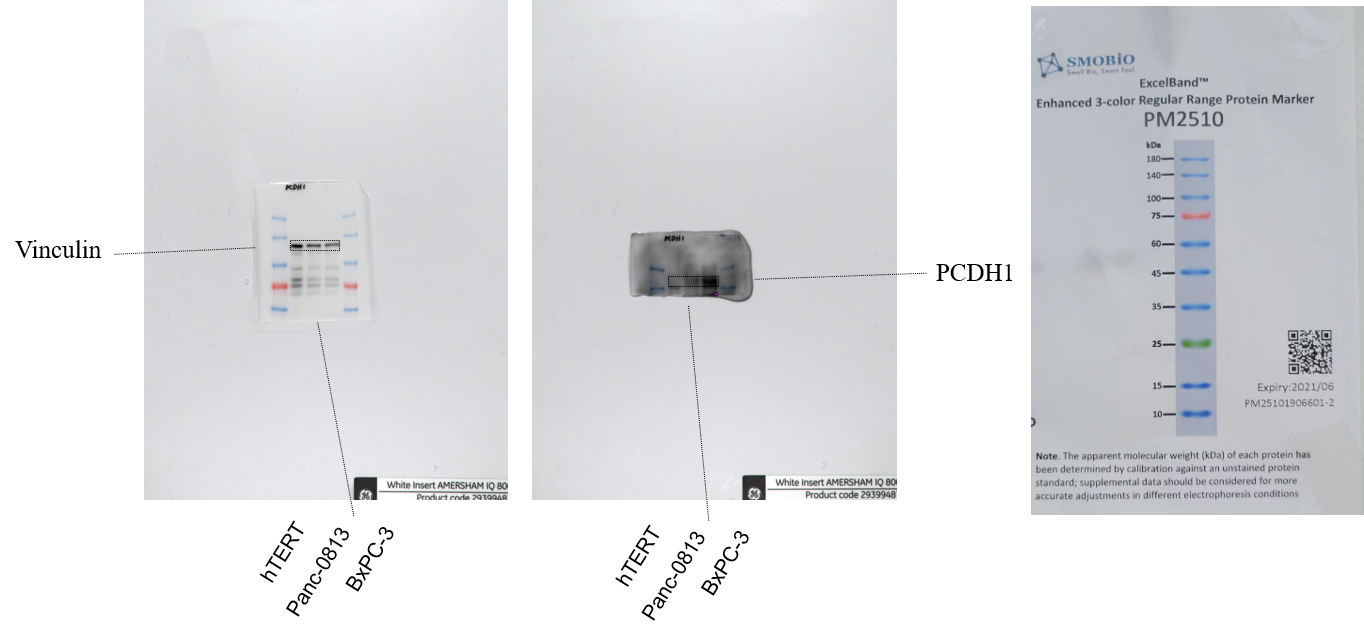


**Fig. S2.**


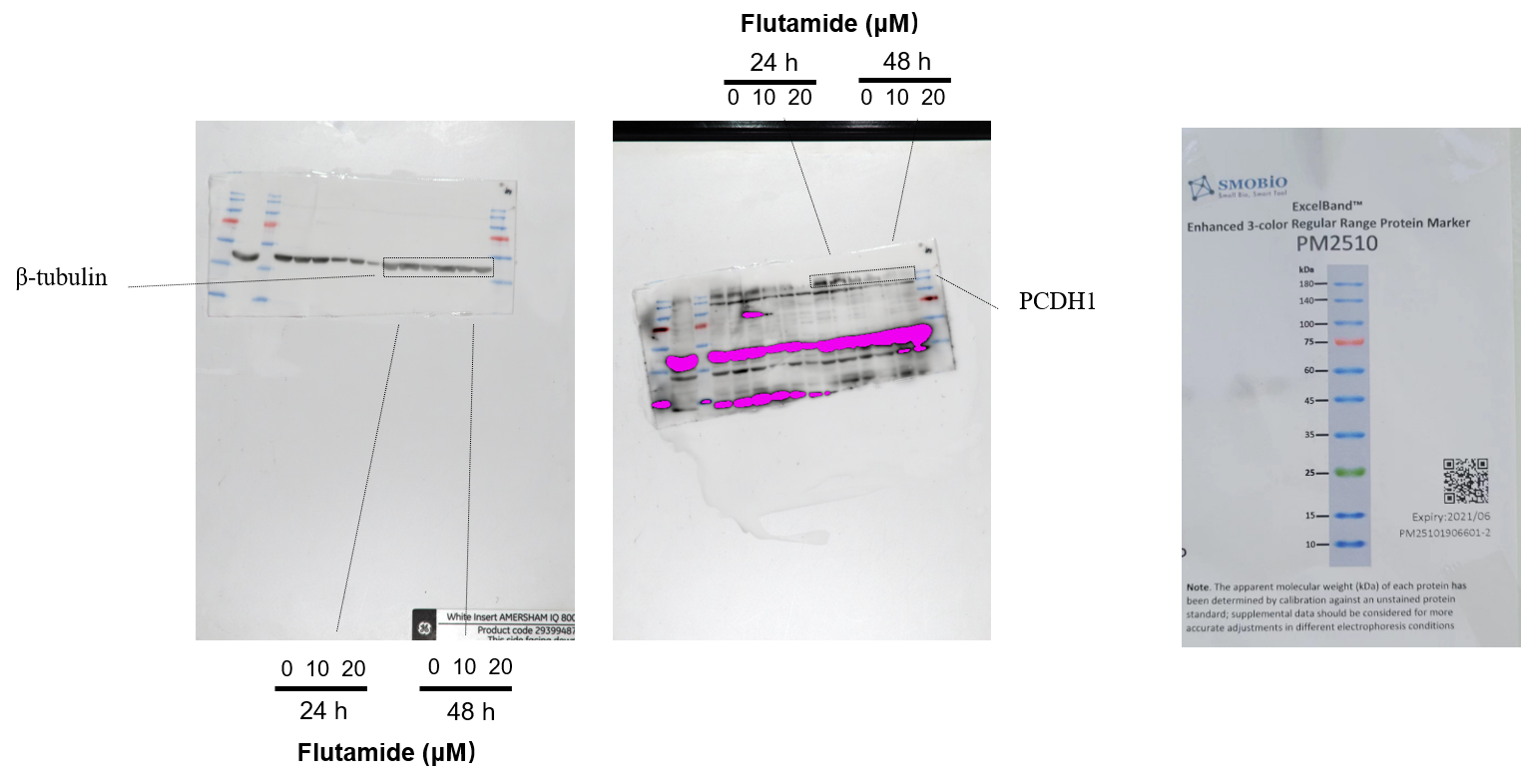

Supplement: Supplementary file 2 — Supplementary Material 2 [file 12885_2023_11474_MOESM2_ESM.docx]
